# Clinical evaluation of a web-based personalized recommendation system with electronic health record interface to optimize healthcare resources during SARS-CoV-2 surges

**DOI:** 10.1038/s41598-023-48325-9

**Published:** 2023-12-15

**Authors:** Alexander Henry Thieme, Maximilian Gertler, Brar Christian Piening, Friederike Maechler, Justus Benzler, Claudia Hartmann, Peter Heumann, Joachim Seybold, Valerie Kirchberger, Volker Budach, Frank Mockenhaupt, Mirja Mittermaier

**Affiliations:** 1 Department of Medicine and Biomedical Data Science, Stanford University, Stanford, CA 94305 USA; 2 Department of Radiation Oncology, Charité - Universitätsmedizin Berlin, Corporate Member of Freie Universität Berlin and Humboldt-Universität zu Berlin, Augustenburger Platz 1, 13353 Berlin, Germany; 3 Berlin Institute of Health (BIH), 10178 Berlin, Germany; 4 Charité Centre for Global Health, Institute of International Health, Charité - Universitätsmedizin Berlin, Corporate Member of Freie Universität Berlin and Humboldt-Universität zu Berlin, Berlin, Germany; 5 Institute of Hygiene and Environmental Medicine, Charité - Universitätsmedizin Berlin, Corporate Member of Freie Universität Berlin and Humboldt-Universität zu Berlin, Berlin, Germany; 6 Robert Koch Institut, Berlin, Germany; 7 Department of Psychosomatic Medicine, Charité - Universitätsmedizin Berlin, Corporate Member of Freie Universität Berlin and Humboldt-Universität zu Berlin, Berlin, Germany; 8 Department of Clinical Procedures GB-IT, Charité - Universitätsmedizin Berlin, Corporate Member of Freie Universität Berlin and Humboldt-Universität zu Berlin, Berlin, Germany; 9 Charité - Universitätsmedizin Berlin, Corporate Member of Freie Universität Berlin and Humboldt-Universität zu Berlin, Berlin, Germany; 10 HRTBT Medical Solutions GmbH, Berlin, Germany; 11 Department of Infectious Diseases, Respiratory Medicine and Critical Care, Charité - Universitätsmedizin Berlin, Corporate Member of Freie Universität Berlin and Humboldt-Universität zu Berlin, Berlin, Germany

**Keywords:** Population screening, Risk factors, Data acquisition, Data integration, Diagnosis

## Abstract

During the SARS-CoV-2 pandemic, the German healthcare system faced challenges of efficiently allocating testing resources. To address this, we developed an open-source personalized recommendation system (PRS) called “CovApp”. The PRS utilized a questionnaire to estimate the risk of infection, provided personalized recommendations such as testing, self-isolation, or quarantine, and featured QR code data transmission to electronic health records. The PRS served up to 2.5 million monthly users and received 67,000 backlinks from 1800 domains. We clinically evaluated the PRS at the SARS-CoV-2 testing facility at Charité and observed a 21.7% increase in patient throughput per hour and a 22.5% increase in patients per day. Patients using the PRS were twice as likely to belong to the High Risk group eligible for testing (18.6% vs. 8.9%, p < 0.0001), indicating successful compliance with CovApp’s recommendations. CovApp served as a digital bridge between the population and medical staff and significantly improved testing efficiency. As an open-source platform, CovApp can be readily customized to address emerging public health crises. Further, given the EHR interface, the app is of great utility for other applications in clinical settings.

## Introduction

The severe acute respiratory syndrome coronavirus 2 (SARS-CoV-2), causing coronavirus disease 2019 (COVID-19), is highly contagious^1, 2^ and can spread from symptomatic and asymptomatic carriers^3, 4^. Due to the exponential spread of the virus and the broad clinical spectrum of symptoms, the German healthcare system was challenged by a large demand for laboratory tests to confirm the diagnosis of SARS-CoV-2 during the first surge of the pandemic. This was accompanied by uncertainty among the population, which led to a high amount of consultations at testing facilities of patients that did not fulfill the criteria to receive testing. Besides limited laboratory test capacity, throughput at testing facilities was a bottleneck. We identified that inquiring about symptoms and asking about travel and medical history occupied much time for the medical staff. Thus, an electronic health history questionnaire that enabled importing of the collected data into an electronic health record (EHR) to save resources while meeting data privacy laws was expected to speed up the processes significantly.

Furthermore, the population sought reliable information about the new virus and appropriate safety measures. However, despite the need for information about social distancing, hygiene, “when to test”, “when and how to self-isolate”^5, 6^, “who has a high risk for a severe course of disease”, and “who has a high risk for an infection”, not much easily understandable and case-individual information was available during the first surge of the SARS-CoV-2 pandemic.

We identified that informing the population and providing an electronic entry point for patients into the healthcare system played a pivotal role in the pandemic to allow case-based recommendations, resulting in efficient usage of healthcare infrastructures. We, therefore, developed a web-based personalized recommendation system (PRS) named CovApp (links to the source code in Code Availability) which could be used by patients for self-assessment of their individual risk of a SARS-CoV-2 infection, severe disease, and secondary infections using their own device such as a smartphone, even before the first contact to the health system was made. The PRS was developed with scalability in mind to be deployed nationwide. Ease-of-use, simple data transfer to an EHR, and a minimalistic user interface were primary goals during development to lower barriers to usage.

## Results

**Figure 1:**
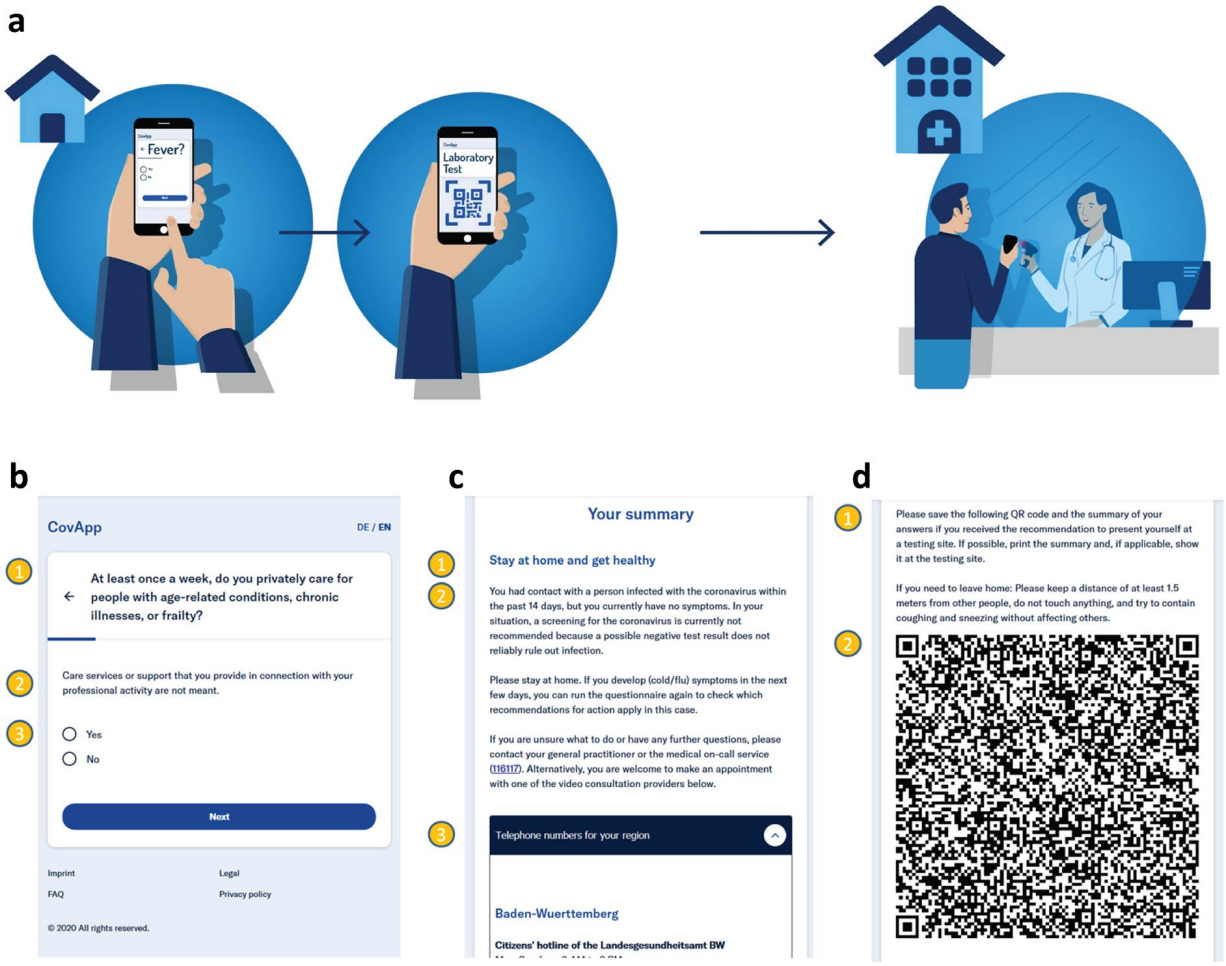
Working principle and screenshots of the PRS CovApp. (**a**) A person suspecting a SARS-CoV-2 infection can complete a questionnaire at home, get a recommendation based on his/her answers and current guidelines and speed up the admission procedure at the testing facility by allowing the medical staff protected behind a glass window to scan the QR code from the smartphone’s display. (**b,c**) Screenshots of CovApp—The user interface has been intentionally reduced to its simplest form for maximal user acceptance and low barriers for usage: (**b**) Question dialog with (1) the question, (2) a comment further explaining the question and (3) the answer options, (**c**) recommendations screen with (1) headline of recommendations, (2) detailed explanation and (3) information regarding telephone hotlines and video consultation and (**d**) bar code screen with (1) instructions for usage and minimizing the risk of infection of others, and (2) the QR code which can be scanned on-site at the hospital to speed up the testing procedure.

**Figure 2:**
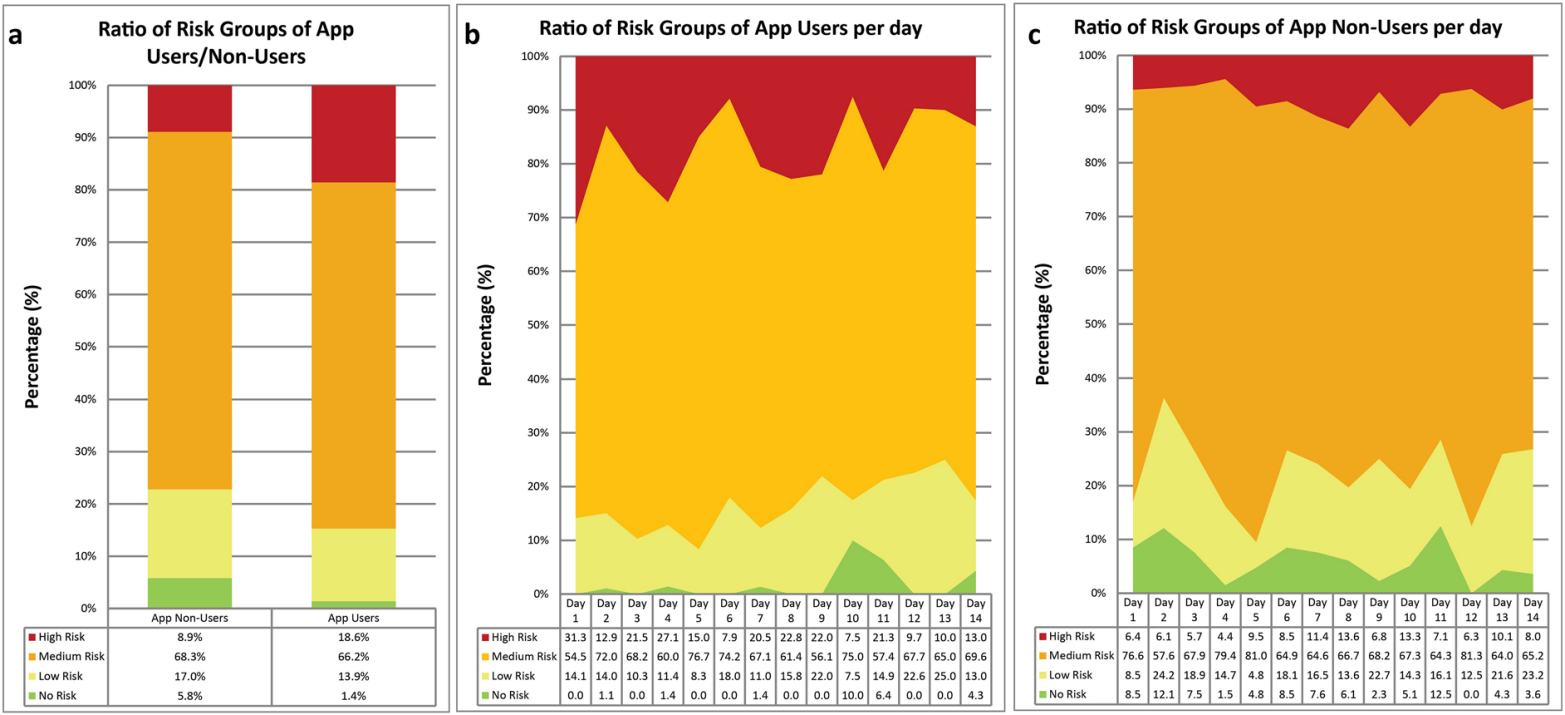
Risk groups of the app and non-users. The ratio of risk groups of (**a**) app users and non-users during the observed time interval (March 18th till 31st, 2020), and (**b**) app users, and (**c**) app non-users per day. Patients who used the app belonged significantly more frequently to the High Risk group and less frequently to lower risk groups, which suggests an effective preselection mechanism by the app (p<0.0001).

Between March 3rd and March 31st, 2020, 3945 patients were tested for SARS-CoV-2 at the testing facility of Charité—Universitätsmedizin Berlin. Patient characteristics are displayed in Table 1. Legal and data protection approvals were granted. The PRS was locally launched on March 12th, 2020, at the Charité testing site, and the information on whether a patient used the app was stored in the EHR beginning March 18th. The questionnaire assessed the individual risk of infection (Table 2, Fig. 1). On the basis of the users’ answers, personalized recommendations were given (e.g., No Risk group—no contact, no symptoms, and thus no test recommended). Based on these recommendations, patients were pre-selected (test necessary or not), aiming to reduce the number of unnecessary patient contacts at the testing facility. Further, the patients’ answers could be transferred via a QR code into the hospital’s EHR system. The throughput of patients per hour at our testing facility was significantly higher after the introduction of the PRS (median 13.39, IQR [12.3–13.8] vs. 11.0, IQR [10.1–11.4] patients per hour, p = 0.0068), which corresponds to a relative increase of 21.7% (Table 3). The number of patients per day was higher as well (median 149.5, IQR [135–164.5] vs. 122, IQR [116–139] patients per day, p = 0.03), with a relative increase of 22.5% (Table 3). After March 18th, the PRS was used by 873 (56.8%) of 1857 patients (Table 1). On March 23rd, 2020, a partial lockdown was implemented. When comparing PRS users with PRS non-users, PRS users were younger (median age 34, IQR [27–44]; vs. non-users: median age 37, IQR [28–49], p < 0.0001), more frequently symptomatic, and more often in contact with a known COVID-19 case than non-users (Table 1). Although the PRS users were younger, the proportion of pre-existing conditions (comorbidities) was comparable (Table 1), while typical COVID-19 symptoms such as dyspnea, fever, and exhaustion were higher in the PRS user group (Table 1), though the symptom cough occurred with comparable frequency. We did not observe a difference in sex ratio between PRS users and non-users. We observed a higher proportion of High Risk patients in the PRS user vs. non-user group (18.6% vs. 8.9%, p < 0.0001) and a lower proportion of Medium Risk (66.2% vs. 68.3%), Low Risk (13.9% vs. 17.0%) and No Risk (1.4% vs. 5.8%) patients (Fig. 2).

**Table 1:** Patient characteristics.

Parameter (date)	All patients (3–31 March)	PRS users (18–31 March)	PRS non-users (18–31 March)	p-value
n	3944	873	984	
Age, median (IQR)	34 (26–46)	34 (27–44)	37 (28–49)	< 0.0001
Sex male, % (n)	49.5 (1951)	49.1 (429)	49.2 (484)	0.9999
Comorbidities, % (n)				
Chronic lung disease	9.0 (353)	11.8 (103)	9.5 (93)	0.2174
Chronic heart disease	3.3 (130)	5.3 (46)	5.6 (55)	0.5226
Diabetes	4.1 (160)	2.5 (22)	3.7 (36)	0.1035
Obesity	2.8 (110)	4.9 (43)	4.6 (45)	0.8871
Symptoms at consultation day				
No symptoms	11.0 (435)	1.6 (14)	9.0 (89)	0.6511
Direct contact to positive person	35.8 (1411)	48.8 (426)	37.5 (369)	< 0.0001
Dyspnea	13.0 (513)	36.1 (315)	13.9 (137)	< 0.0001
Fever	26.5 (1045)	31.5 (275)	19.5 (192)	< 0.0001
Chest pain	6.1 (240)	6.1 (53)	11.4 (112)	0.018
Chills	21.6 (851)	33.4 (292)	23.9 (235)	< 0.0001
Physical exhaustion	46.2 (1823)	77.8 (679)	43.2 (425)	< 0.0001
Body aches	29.3 (1156)	40.0 (402)	29.0 (285)	< 0.0001
Cough	59.7 (2354)	60.3 (526)	58.8 (579)	0.5386
Runny nose	40.0 (1576)	60.7 (530)	39.9 (393)	< 0.0001
Diarrhoea	13.0 (511)	25.7 (224)	14.4 (142)	< 0.0001
Sore throat	48.1 (1897)	64.6 (564)	50.3 (495)	< 0.0001
Headache	41.7 (1645)	66.2 (578)	45.0 (443)	< 0.0001

The study was conducted between March 3rd and March 31st, 2020. The left column displays all patients within the study period. CovApp was introduced on March 12th, 2020, and on March 18th, the information on whether a patient used the PRS or not was stored in the EHR system. The two columns in the middle display the patient’s characteristics of PRS users vs. non-users between March 18th and March 31st. The right column displays the p-values.

**Table 2:** CovApp questionnaire.

No.	Category	Question	Answer options
1.	Group 1: Social environment	What is your current living situation?	1. Living alone 2. Living together with family, in a shared flat, or in a supervised community facility
2.		At least once a week, do you privately care for people with age-related conditions, chronic illnesses, or frailty?	1. Yes 2. No
3.		Do you work in one of the following areas?	1. In the medical field 2. In a community facility (school, day care center, university, home etc.) 3. No, in none of the above
4.	Group 2: risk factors for SARS-CoV-2 infection	Have you had close contact with a confirmed case?	1. Yes 2. No
5.		What day was the last contact?	1. Date
6.		Have you had a fever (over 38 °C) in the past 24 h?	1. Yes 2. No
7.		Have you had a fever (over 38 °C) in the past 4 days?	1. Yes 2. No
8.		In the past 24 h, have you had chills?	1. Yes 2. No
9.		Which of the following symptoms have you had in the past 24 h? (Multiple selection possible)	1. Feeling tired or weak 2. Body aches 3. Diarrhea 4. Headache
10.		In the past 24 h, have you had a persistent cough?	1. Yes 2. No
11.		In the past 24 h, have you had a runny nose?	1. Yes 2. No
12.		In the past 24 h, have you had a sore throat?	1. Yes 2. No
13.		In the past 24 h, did you feel that you were more quickly out of breath than usual?	1. Yes 2. No
14.		With regard to all questions about symptoms: since when have you had the symptoms you specified?	1. Date
15.	Group 3: risk factors for severe Covid-19 disease progression	How old are you?	1. Under 40 2. 40–50 3. 51–60 4. 61–70 5. 71–80 6. Over 80
16.		Are you 65 years old or older?	1. Yes 2. No
17.		Have you been diagnosed with chronic lung disease by a doctor?	1. Yes 2. No 3. I don’t know
18.		Have you been diagnosed with diabetes by a doctor?	1. Yes 2. No 3. I don’t know
19.		Have you been diagnosed with heart disease by a doctor?	1. Yes 2. No 3. I don’t know
20.		Have you been diagnosed with adipositas (obesity) by a doctor?	1. Yes 2. No 3. I don’t know
21.		Are you currently taking steroids?	1. Yes 2. No 3. I don’t know
22.		Are you currently taking immunosuppressants?	1. Yes 2. No 3. I don’t know
23.		Have you been vaccinated against flu between October 2019 and today?	1. Yes 2. No
24.		Do you smoke?	1. Yes 2. No
25.		Are you pregnant?	1. Yes 2. No 3. I don’t know

*No.* number.

**Table 3:** The table shows the number of patients per day at the Charité - Universitätsmedizin Berlin COVID-19 testing site, the opening/working hours of the testing site per day, and the average number of patients per hour.

Date	Patients per day	Working hours	Patients per hour
3-Mar-2020	116	10.22	11.35
4-Mar-2020	113	11.13	10.15
5-Mar-2020	119	10.82	11.00
6-Mar-2020	123	11.98	10.26
7-Mar-2020	122	13.02	9.37
8-Mar-2020	95	10.00	9.50
9-Mar-2020	151	12.03	12.55
10-Mar-2020	139	12.32	11.29
11-Mar-2020	173	12.10	14.30
12-Mar-2020*	178	11.22	15.87
13-Mar-2020	172	14.25	12.07
14-Mar-2020	166	12.73	13.04
15-Mar-2020	134	9.83	13.63
16-Mar-2020	130	12.20	10.66
17-Mar-2020	156	11.05	14.12
18-Mar-2020	147	10.55	13.93
19-Mar-2020	129	9.60	13.44
20-Mar-2020	165	11.58	14.24
21-Mar-2020	138	11.57	11.93
22-Mar-2020	126	10.00	12.60

On March 12th, 2020, the PRS was introduced at the Charité - Universitätsmedizin Berlin COVID-19 testing site. After introduction of the PRS, the efficiency at the testing site increased, and as a result, the average number of patients per hour went up.

*Date of first usage of CovApp.

On March 18th, 2020, the PRS was announced to the general German public by a press release of the Charité - Universitätsmedizin Berlin. The PRS was well received and estimated to be used by more than 2.5 million users in the first month after release from website traffic. At its peak, the PRS received 67,400 backlinks from 1800 linking domains. The PRS was recommended to citizens by major authorities such as the Robert Koch-Institute (German National Public Health Institute), the German Federal Ministry of Health, and Federal Centre for Health Education. Multiple hospitals found the approach of the PRS useful and requested additional information to implement the data import from the PRS’s QR code. We, therefore, decided to create a development documentation and released the source code as open source via a publicly accessible GitHub repository^7^. After our successes in Germany, we released the PRS in the United States^8^.

## Discussion

Here, we demonstrated that the PRS (1) was scalable to a larger proportion of the general population, (2) provided personalized recommendations that patients complied to, and (3) accelerated clinical processes by evaluating and transferring anamnestic information to the EHR and guiding patients before their consultation at a testing facility.

At the beginning of the pandemic, testing capacity was very limited, and only patients with a high probability of being infected were eligible for testing. Strict pre-selection of patients was considered pivotal to prevent the overloading of healthcare infrastructures. Additionally, time-consuming paperwork and the acquisition of medical history, along with a shortage of medical staff at testing sites, limited the number of patients that could be tested.

Patients who were tested positive for SARS-CoV-2, might have already infected other close contacts before they started their quarantine. To identify these contacts as quickly as possible in order to interrupt this transmission chain, several other research groups developed digital tools, namely contact tracing tools^9, 10^, as part of containment strategies.

During the first surge, the PRS fulfilled the need of 2.5 million users to assess their personal risk of a SARS-CoV-2 infection and to get a personalized recommendation for further actions. Consequently, the PRS provided personalized information supporting public health measures such as self-isolation and social distancing^5, 6, 11^. At the testing facility of Charité—Universitätsmedizin Berlin, we could observe further benefits of the PRS such as the touchless transmission of patient answers using QR code technology and the acceleration of medical history-taking as fewer questions had to be asked by the medical staff^12^. We observed a significantly higher patient throughput and number of daily patients after the introduction of the PRS. We observed an increased proportion of individuals in the High Risk group and a reduced proportion of individuals with a Medium and Low Risk for an infection among the PRS users. This could indicate an effective pre-selection mechanism where users based their decision to attend the testing facility on the personalized recommendations of the PRS. Potential other influences on the patient throughput, such as improving medical staff routine, were not accounted for in the analysis, as it was not possible to measure these, given the dynamic of the situation. Some differences between PRS users vs. non-users (e.g., regarding age, Table 1) might be related to differences in smartphone usage^13, 14^. Also, whether the same effect can be observed at a larger scale in multiple institutions still needs to be demonstrated.

An important requirement for usage of the PRS was access to and familiarity with an internet-enabled device. From microcensus conducted by the Federal Statistical Office for Germany it is known that there was a significant difference in daily internet usage of persons older than 65 years vs. younger^15^. Other representative statistics saw a decreased usage of smartphones in the older population (64.5% for 70 years and older vs. 89.4% to 99.4% for those aged between 14 and 69 years)^13^. Indeed, in this study we noticed a less frequent usage of the PRS in patients older than 60 years. Patients older than 60 years belong to the risk group for severe COVID-19 disease progression and have a significantly increased risk of death^16–18^. Consequently, CovApp might have a limited reach within this important COVID-19 risk group. This could be addressed by providing other frontends to the PRS besides the web interface, e.g. systems that use text-to-speech and voice recognition as user interface, to eliminate barriers and make the technology available to older patients^19^.

In retrospect, we identified several key factors for the successful development and implementation of CovApp. Firstly, leveraging patients' smartphones for access to the PRS via a static web app enabled rapid scalability and cost savings. This approach efficiently delivered content to a vast user base, with the more resource-intensive computations handled by the users' devices. Secondly, we found that a concise yet precisely worded questionnaire balanced accuracy with simplicity, fostering user acceptance. Moreover, the technology's agility in adapting to evolving scenarios was crucial, especially because some users might have relied on the PRS as their only source for information. To facilitate this, our team developed and open-sourced a software named "CovQuestions" to update the questionnaire, including risk assessment logic and recommendations (link to the source code in Code Availability).

Before developing the app, we identified with clinicians and nurses the most time-consuming tasks where the PRS could best help saving the staff’s time. Here, taking a patient’s history and checking if a patient was eligible for testing was identified as very time-consuming. Programming the interface to the hospital’s EHR required some time. Thus, we recommend developing the content of the PRS simultaneously, as we did. From our perspective, the PRS became widely accepted by both patients and healthcare professionals because it addressed the most urgent questions of the population in a concise, precise and straightforward language while significantly reducing staff time at the hospital.

Furthermore, due to the high number of users, a vast amount of data could be generated by this service. Later versions of the PRS offered the possibility for users to voluntarily donate their answers to a research database including information regarding an approximate location. Therefore, the PRS could be an important source for big data. Recently, the PRS became part of an early warning system, which combines symptoms, GPS, genomic and weather data to detect local outbreaks and predict their temporal and spatial evolution of new infections.

## Conclusion

The PRS was deployed nationwide during the first surge of the SARS-CoV-2 pandemic in Germany and was used 2.5 million times within the first month. The PRS served as a self-assessment and information tool for patients suspecting a SARS-CoV-2 infection and could transfer the collected information to an EHR. An increased patient throughput was observed at the testing facility of Charité—Universitätsmedizin Berlin by using QR code technology to transmit patient answers digitally and to speed up the medical history-taking process. At our testing facility, we observed that significantly more PRS users belonged to the High Risk group and less to lower risk groups suggesting that users followed the personalized recommendations resulting in a better patient pre-selection. The PRS could be used to better utilize healthcare resources and steer population behavior toward appropriate prevention, diagnostics, and care.

## Methods

### Development of the personalized recommendation system

An interdisciplinary group consisting of clinicians, IT experts, and administrative staff aimed to develop a PRS with the following requirements: (1) easy-to-use interface, (2) personalized recommendations and instructions based on patient answers, (3) digital transfer of patient answers to an EHR, (4) compliance with the European General Data Protection Regulation (GDPR), (5) easily scalable computing capacity at low cost, (6) and compliance with the Act on Medical Devices (MPG). To shorten the development time, an existing tool to assess electronic patient-reported outcomes for cancer patients created at the Department for Radiation Oncology, Charité—Universitätsmedizin Berlin, was used as the backbone^20^. The decision was made to implement the PRS as a static web app, so that any web-enabled device independent of the operating system could connect to the service. A responsive design adapted the layout of the PRS to different screen sizes ranging from smartphones to tablets and personal computers. Common web standards were used to implement the dynamic display and evaluation of questions on the user’s device, e.g., HTML5^21^, JavaScript^22^, and JavaScript Object Notation^23^ (JSON). Risk assessment and personalized recommendations were implemented as a logic tree which was evaluated on the end user’s device, thus leveraging its computing power to allow for high scalability at low costs. A solution for the digital transfer of the data and import to an EHR represented a real challenge. Transferring the data directly from the end user’s device to the EHR was not feasible, as this approach would have added complex legal requirements and security measures for the exchange of health data. Also, clinical computer systems typically reside inside a protected network, complicating data exchange with patient devices using the public internet. Therefore, a technical approach was sought without the necessity to transfer data using the internet. A method was developed that stored patient answers in a Quick Response (QR) code^24^ that can be scanned from the patient’s smartphone or a print-out at the hospital upon admission. The data could be imported into the electronic patient records without the need for a network link between the patient device and the hospital system. Thus, the data was stored on the patient’s device until the consent for the transfer of the data is given by the patient presenting the QR code to the medical staff for scanning. This approach is compliant with GDPR rules resulting in GDPR certification of the PRS.

The data stored inside the QR code used the Extensible Markup Language^25^ (XML) format with the support of many medical vendors. Additionally, we used the highest error correction scheme of QR code (Level H with 30% redundancy) to enable scanning of the code through a closed glass window which is necessary to avoid contact between the patient and healthcare workers and to increase occupational health and safety.

Importing the data stored in the QR code into the EHR of Charité—Universitätsmedizin Berlin (SAP Enterprise R3, Version 470, SAP SE, Walldorf, Germany) was implemented by a scanner with a keyboard wedge interface that translated the information stored in the code into keyboard strokes. The information was entered into a textbox which was then decoded into a human-readable form.

In the testing procedure, all major browsers (Chrome, Firefox, Edge, Internet Explorer) in their current version and 2 previous versions, and all major operating systems (Windows, Android, iOS) were tested on multiple devices such as smartphones, tablets, and notebooks/PCs with different screen sizes to maximize the usability of the PRS.

### Questionnaire

The questionnaire was provided bilingual in English and German with the possibility to add further languages. The questionnaire was intended to be answered by the respondent without additional assistance from medical staff.

#### Questions

The questionnaire consisted of 25 questions, grouped into three categories: 1. social environment of the patient to assess the relevance of exposure and potential of spreading, 2. clinical symptoms of the potential SARS-CoV-2 infection, and 3. risk factors for progression to a severe course of COVID-19 (Table 2). Two different question types were used: (1) twenty-three multiple choice questions, (2) two questions regarding a date. This greatly reduced the complexity of the questionnaire and allowed for efficient storage of the answers in the QR code. Each question was presented in a simple dialog window with the question on the top, an explanation of the question below, and multiple answer options or a field to enter a date (Fig. 1a,b). Each question required an answer. For some questions, the answer option “I don’t know” was offered. A branching logic was implemented, e.g. if no symptoms were reported, the question regarding the date for onset of symptoms was not asked. Despite the simplicity of this question structure, all versions of the German national recommendations for SARS-CoV-2 testing developed by the Robert Koch-Institut (RKI) could be integrated^26^. The simple structure allowed for swift adjustments of the questionnaire to adapt to changes in these recommendations, which were necessary multiple times in March 2020 e.g., due to changes (and eventually removal) of risk areas by the RKI^27^.

#### Risk assessment

**Figure 3:**
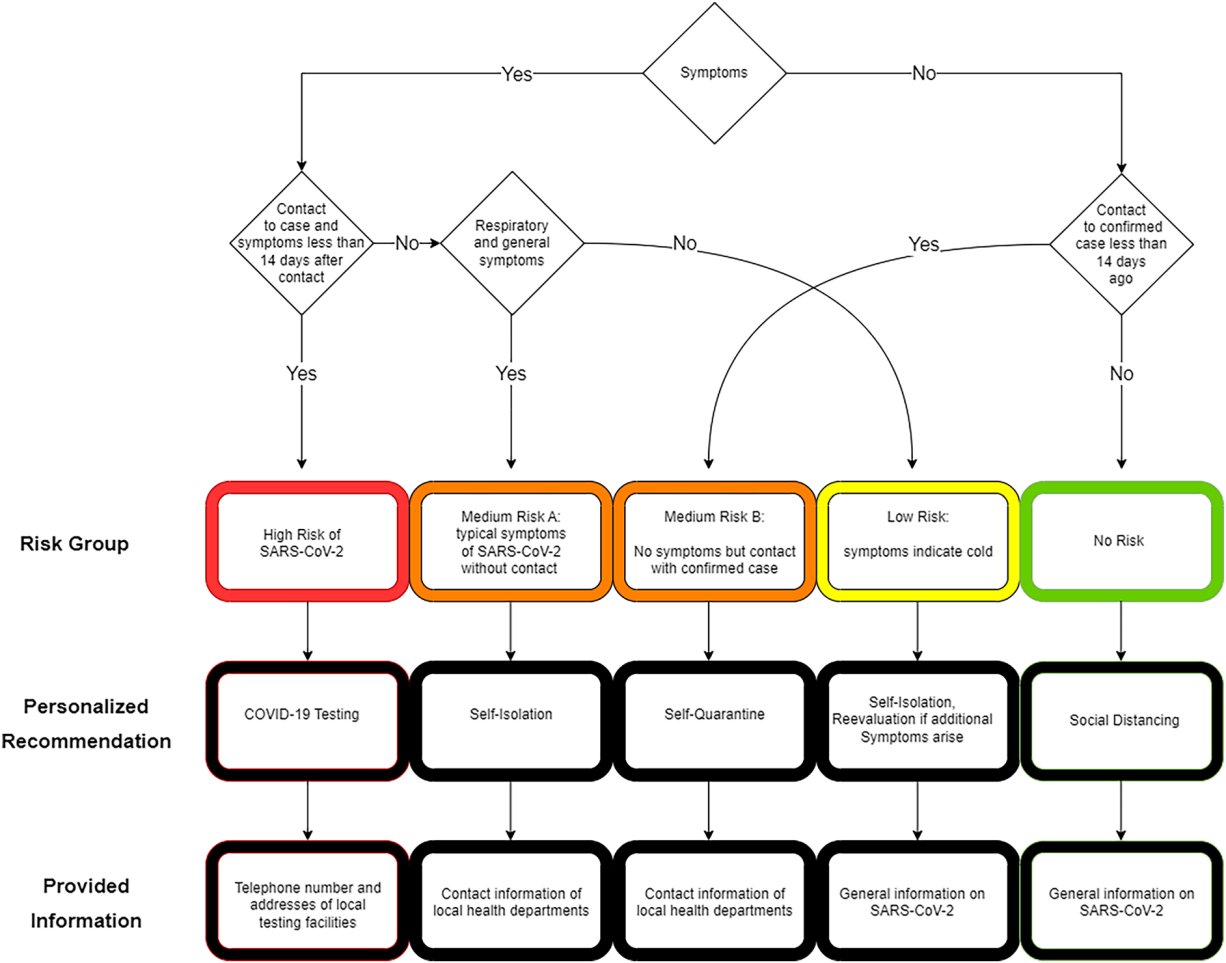
Diagram for the decision tree used by the PRS. The diagram displays how the PRS assessed the risk group based on patient answers. Based on reported symptoms and contact history, a risk group was assigned. “Personalized recommendations” and the related “provided information” were provided in accordance with the assigned risk group. Table 2 displays the “CovApp Questionnaire”, including the questions that were used to identify a patient’s symptoms.

The individual risk of infection was assessed on the basis of the user’s answers in group 2 of the questionnaire (Table 2, Fig. 3). Evaluation of answers and application of the logic was performed on the user’s device. The PRS distinguished five different risk groups: (1) High Risk: patients who had contact with a confirmed case and onset of respiratory and general symptoms within 14 days of contact, (2) Medium Risk A: patients without contact history but with a combination of respiratory and general symptoms, Medium Risk B: patients who had contact with a confirmed case without symptoms, (3) Low Risk: patients with either respiratory or general symptoms, (4) No Risk: patients with no contact history and no symptoms. Based on the estimated risk, users received personalized recommendations, contact details to local medical facilities, and further information sourced from the German public health institute (Fig. 3).

#### Personalized recommendations

The personalized recommendations were based on the assessed risk and included diagnostic options, isolation/quarantine, and advice on seeking medical care (Fig. 3). Known risk factors for progression to severe forms of COVID-19 and death were chronic diseases^16, 28^ and cancer^29^. The personalized recommendations (Fig. 1c) were presented after the last page of the questionnaire (Fig. 1b) and consisted of a headline and a detailed explanation of the personalized recommendations and instructions for the next steps (Fig. 1c). Testing was recommended to all users with High Risk or the presence of respiratory symptoms if the patient belonged to the risk group for severe illness or worked in the medical domain. For Medium and Low Risk, self-isolation was recommended. For patients in the No Risk group, only general information for social distancing and hygiene was given.If a patient had shortness of breath, the PRS recommended immediate medical advice regardless of risk group allocation. Specific information was displayed for e.g. healthcare professionals or users with intense social contacts.

#### Connecting with the healthcare system

If laboratory testing was recommended, the PRS provided information on establishing contact with the healthcare system (Fig. 1c). The user was given the choice of a phone call or video consultation. If a phone call was preferred, the user was presented with the central national hotline for SARS-CoV-2 and additionally with local hotlines per state. For laboratory testing, the user could use the QR code at the testing facility to speed up the testing procedure (Fig. 1d).

#### Patient cohort and clinical evaluation of the personalized recommendation system

Patient data were retrospectively evaluated from March 3rd, i.e., the opening of the testing site, to March 31st, 2020. The study was approved by the ethics committee of the Charité—Universitätsmedizin Berlin (EA4/083/20) and was performed according to the Declaration of Helsinki and Good Clinical Practice principles (ICH 1996). As this is a retrospective study, the need of informed consent was waived by the ethics committee of the Charité—Universitätsmedizin Berlin (EA4/083/20).

Starting March 12th, the PRS was promoted by a public press release, medical staff, and posters installed in the waiting area at the local testing facility of the Charité—Universitätsmedizin Berlin.

The following information was extracted from electronic patient records: Age, sex, symptoms in the last 24 h (yes/no), general symptoms in the last 24 h (yes/no), respiratory symptoms (yes/no), contact history with a confirmed SARS-CoV-2 case (yes/no), chronic diseases (yes/no for lung, heart, diabetes and other chronic diseases), PRS usage (yes/no, since 18th March 2020 stored in medical records), risk group (No Risk, Low Risk, Medium Risk, High Risk as previously defined), number of patients per day, treatment time of the first patient, treatment time of last patient. The throughput of patients per day and per hour of the testing facility before and after the implementation of the PRS was calculated. The number of medical staff involved in the admission procedure of patients stayed the same, and no other adjustments were made. For patient throughput evaluation, data between March 3rd till 22nd was used. Afterward, collected data were not used to exclude a possible bias due to a partial lock-down implemented by the government on March 23rd 2020. On March 12th, the CovApp was introduced at the testing site, and data between March 18th and 31st were used to compare PRS users and non-users with regard to the proportions of the four risk groups. Website traffic was analyzed and evaluated with Matomo (version 3.13.3, open-source software, www.matomo.org). The number of referencing domains and backlinks was evaluated using the online tool Ahrefs^30^. A Google search was performed to evaluate the number of news articles mentioning the term CovApp.

#### Statistical methods

The median and interquartile ranges (IQR) of throughput of patients per day and per hour at the local testing facility at Charité—Universitätsmedizin Berlin before and after implementation of the PRS were calculated and the rank sums before and after implementation compared non-parametrically with the Mann–Whitney-U test. The proportions of risk groups of PRS users vs. non-users were compared with a Chi-squared test. All tests were 2-sided with an alpha level of 0.05. The R environment for statistical computing^31^ (version 3.4.7, R Foundation, Vienna, Austria) was used for data processing and statistical analyses.

## Data Availability

The data that support the findings of this study are available from the corresponding author upon reasonable request.

## Code availability

The source code of CovApp 1.0 was released as open source and made publicly accessible at GitHub (https://github.com/CovOpen/CovApp-1.0). The code was made available under the General Public License (GPL). The source code of CovApp 2.0 was released as open source and made publicly accessible at GitHub (https://github.com/CovOpen/CovApp-2.0). The code was made available under the MIT License. The source code of CovQuestions was released as open source and made publicly accessible at GitHub (https://github.com/CovOpen/CovQuestions) under the Apache License 2.0.
